# Alterations of large‐scale functional network connectivity in patients with infantile esotropia before and after surgery

**DOI:** 10.1002/brb3.3154

**Published:** 2023-07-11

**Authors:** Jianlin Guo, Yuanyuan Chen, Wen Liu, Lijuan Huang, Di Hu, Yanqiu Lv, Huiying Kang, Ningdong Li, Yun Peng

**Affiliations:** ^1^ Department of Radiology, MOE Key Laboratory of Major Diseases in Children Beijing Children's Hospital, Capital Medical University, National Center for Children's Health Beijing P. R. China; ^2^ Tianjin International Joint Research Center for Neural Engineering Academy of Medical Engineering and Translational Medicine, Tianjin University Tianjin P. R. China; ^3^ Department of Ophthalmology Beijing Children's Hospital Capital Medical University, National Center for Children's Health Beijing P. R. China; ^4^ Department of Ophthalmology Second Affiliated Hospital of Fujian Medical University Quanzhou P. R. China; ^5^ Key laboratory of Major Diseases in Children Ministry of Education Beijing P. R. China

**Keywords:** infantile esotropia, network connectivity, postoperative, preoperative, visual

## Abstract

**Background:**

Growing evidences have indicated neurodevelopmental disorders in infantile esotropia (IE). However, few studies have analyzed the characteristics of large‐scale functional networks of IE patients or their postoperative network‐level alterations.

**Methods:**

Here, individuals with IE (n = 32) and healthy subjects (n = 30) accomplished the baseline clinical examinations and resting‐state MRI scans. A total of 17 IE patients also underwent corrective surgeries and completed the longitudinal clinical assessments and resting‐state MRI scans. Linear mixed effects models were applied for cross‐sectional and longitudinal network‐level analyses. Correlation analysis was performed to assess the relationship between longitudinal functional connectivity (FC) alterations and baseline clinical variables.

**Results:**

In cross‐sectional analyses, network‐level FC were apparently aberrant in IE patients compared to controls. In longitudinal analyses, intra‐ and internetwork connectivity were observed with significant alterations in postoperative IE patients compared to the preoperative counterparts. Longitudinal FC changes are negatively correlated to the age at surgery in IE.

**Conclusions:**

Obviously, altered network‐level FC benefiting from the corrective surgery serves as the neurobiological substrate of the observed improvement of stereovision, visuomotor coordination, and emotional management in postoperative IE patients. Corrective surgery should be performed as early as possible to obtain more benefits for IE in brain function recovery.

## Graphical abstract

Our findings indicate that altered network‐level functional connectivity benefiting from the corrective surgery serves as the neurobiological substrate of the observed improvement of stereovision, visuomotor coordination and emotional management in postoperative IE patients. More importantly, corrective surgery should be performed as early as possible to obtain more benefits for IE in brain function recovery. To the best of our knowledge, the present study offers first evidence of early surgery contributing to the brain function recovery of IE, which is instructive for the option of surgery opportunity.

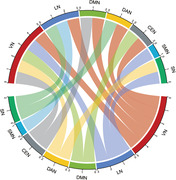

## 1 INTRODUCTION

Strabismus is a common ocular disorder that affects 2–6% of the children, resulting in undesirable consequences including impaired visual acuity, loss of stereoscopic depth perception, poor hand‐eye coordination, and psychosocial disturbance (Suttle et al., 2011; Torp‐Pedersen et al., 2017). Infantile esotropia (IE) is a subtype of concomitant strabismus, which occurs during the first 6 months of life and is distinguished by large‐angle nasal‐ward deviation of both optic axes, cross‐fixation, latent nystagmus, and dissociated vertical deviation (Mehner et al., 2023). Although kinds of theories have been proposed for the etiology of IE, such as impairment in brain regions associated with fusion ability (Yang et al., 2014), the enhanced subcortical ocular motion pathway plasticity (Brodsky, 2019), binocular monocular nasal‐temporal optokinetic asymmetry resulting from the maldevelopment of visual cortex (Brodsky, 2018), the underlying neuropathological mechanism remains controversial and urgently needs to be further explored.

In recent years, functional magnetic resonance imaging (fMRI) has been popular in examining the neurobiological changes of patients with strabismus. As a result, abnormalities of brain structure and function in strabismus patients have been demonstrated through varieties of imaging techniques with different data processing protocols. Specifically, morphometry studies have revealed abnormal gray matter volume (Chan et al., 2004; Su et al., 2022), white matter volume (Ouyang et al., 2017), and cortical thickness (Yin et al., 2021) of some brain regions in patients with strabismus. Altered microstructural properties of white matter implicated in visual pathway have been demonstrated in strabismus patients using voxel‐based analysis (Huang et al., 2016; Yan et al., 2010), tract‐based spatial statistics, and automated fiber quantification analysis of diffusion tensor imaging (DTI) (Duan et al., 2015; Li et al., 2018). More specifically, a voxel‐based DTI analysis has revealed lower fractional anisotropy in middle occipital gyrus and supramarginal gyrus in strabismus adults (Yan et al., 2010); Higher fractional anisotropy values in inferior fronto‐occipital fasciculus and inferior longitudinal fasciculus were apparent in adults with comitant exotropia (Li et al., 2018). An automated fiber quantification study of adults strabismic amblyopia has demonstrated elevated mean diffusivity along several visual‐related fasciculi such as vertical occipital fasciculus, optic radiation, and inferior longitudinal fasciculus (Duan et al., 2015). It has been reported that strabismus patients present abnormal functional connectivity (FC) of the primary visual cortex (Liu et al., 2022; Yan et al., 2019), anomalous interhemispheric FC of visual‐related brain subregions (Peng et al., 2021; Zhang et al., 2021), as well as disrupted spontaneous neural activity of regional homogeneity (Huang et al., 2016; Tan et al., 2022) and amplitude of low frequency fluctuation (He et al., 2021; Hu et al., 2022; Xi et al., 2020) in brain areas related to visual processing. However, few studies have explored the functional alterations of brain in IE. As a rare category of strabismus with congenital fusion deficiency, IE may have uncommon neuroimaging features suggestive of specific neurobiological changes.

Corrective surgery is an essential option in intervention for IE, as alignment of visual axes facilitates the potential for binocularity. Nevertheless, it is worth noting that restoring normal ocular alignment do not promise improvement of stereopsis in some IE patients, and the precise mechanism of which remains perplexing (Çerman et al., 2014; Hug, 2015). Strengthened spontaneous brain activity in visual cortex or microstructural alterations in core components of visuospatial network may account for the stereoscopic vision restoration in postoperative patients with comitant exotropia (Wang et al., 2022; Wu et al., 2022), while no longitudinal studies have investigated the interaction between stereovision recovery and changes in FC in terms of large‐scale networks in IE patients before and after surgery. In addition, controversies concerning the timing of surgery for IE remain difficult to figure out (Mehner et al., 2023). Our previous cross‐sectional research has suggested that, compared to healthy subjects, patients with IE have an aberrant developmental trajectory of FC from birth, exhibiting great potential for correction of compromised neurodevelopment during the early postnatal period, thus, the timely surgical interventions may bring satisfactory benefits for improvements in visual function as well as other visual‐related brain functions (Guo, Chen, Liu et al., 2022a). However, longitudinal studies are necessary for better understanding the effects of surgical intervention on functional recovery of IE during the crucial period of growth and development.

In this study, we analyzed the alterations of large‐scale functional network connectivity using the cross‐sectional sample and what is, as far as we know, the first longitudinal sample of resting‐state fMRI data from patients with IE, with the purpose of investigating whether and how the surgical intervention contributes to the restoration of various brain functions as well as exploring the potential neuropathological mechanism of visual‐related impairment in IE.

## 2 MATERIALS AND METHODS

### 2.1 Participants

At baseline, 32 patients with IE and 30 control participants accomplished the resting‐state fMRI scanning and clinical evaluations. Partial data of cross‐sectional analysis have been reported (the original research includes 17 IE patients and 20 healthy controls) (Guo, Chen, Liu et al., 2022a). The current study recruited additional participants for both IE group and control group and collected new postoperative longitudinal data of IE patients. The diagnosis of IE was based on inward deviation of both eyes with the same degree, onset age of no more than 6 months, and the absence of additional neurological disorders. All patients with IE underwent strabismus surgery the next day after baseline fMRI scans, among whom 17 patients had postoperative follow‐up visits with an average follow‐up period of 11.81 ± 3.04 months. Age‐ and sex‐matched healthy participants were recruited with inclusion criteria as follows: no evidence of ocular diseases, absence of neurological or psychological disorders, and in capable of completing MRI examination. Thus, baseline data of 32 IE patients (mean age: 3.00 ± 1.81, 17 males) and 30 healthy controls (mean age: 3.47 ± 1.76, 16 males) as well as longitudinal data of 17 postoperative patients with IE (mean age: 2.57 ± 2.10, 11 males) were analyzed further. This study was approved by the Medical Research Ethics Committee of Beijing Children's Hospital (approval number: 2022‐E‐093‐Y) and complied with the tenets of the Declaration of Helsinki. The written informed consent was obtained from the parents or legal guardians of the subjects.

### 2.2 MRI data acquisition and preprocessing

Imaging data were acquired with a 3T MRI scanner (General Electric, Discovery MR 750, 8‐channel head coil) in IE patients and healthy participants during natural sleep. To improve the quality of data acquisition and avoid the mixture of MRI data under waking status with those under sleep state, we scheduled the scanning at night when subjects feel sleepy. Once they fall asleep, we transfer them from the preparation room to the scanning bed gently. Earphone and spongy pads were applied to minimize the noise and head motion. A camera facing the scanning bed was employed to monitor the behaviors of subjects. Structural scans contained T1‐weighted three‐dimension brain volume sequence (repetition time [TR]: 8.2 ms, echo time [TE]: 3.2 ms, field of view [FOV]: 256 mm, flip angle: 12°, voxel size: 1 mm^3^) and T2‐weighted imaging (TR: 4538 ms, TE: 93 ms, FOV: 24 cm). Resting‐state fMRI data were collected with echo planar imaging techniques (TR: 2000 ms, TE: 30 ms, flip angle: 90°, FOV: 224 mm, voxel size: 3.5 mm^3^, 240 images). fMRI scanning was performed at baseline and follow‐up visits for IE group in contrast to the single baseline scanning for control participants.

MRI data were preprocessed with scripts based on Analysis of Functional NeuroImages (AFNI, https://afni.nimh.nih.gov/) and FMRIB Software Library (FSL, https://fsl.fmrib.ox.ac.uk/fsl/fslwiki), consisted of removing the first five time‐point images, time correction between slices, head motion correction with linear alignment, scrubbing method for further motion effect control (volumes with framewise displacement bigger than 0.3 mm and no more than three sequential time points were removed), nuisance covariates regression for global signal, white matter signal, cerebrospinal signal and head motion parameters, band‐pass filtering of 0.01–0.1 Hz, spatial smoothing with a 6 mm full width at half maximum Gaussian kernel. Global signal, the averaging signal from all gray matter voxels, contains both the neural and non‐neural (like head motion) components. Therefore, whether to remove the global signal is really dependent on the specific question. First, the global signal removal is good to further control the impact of head motion. Then, the focus of this article is to find and delineate the regional alterations associated with disease, for which it is better to remove the global information and enhance the local information in the blood oxygen level‐dependent signals. Overall, performing global signal correction is helpful to reduce the potential confounding effects and contributes to the detection of regionally specific FC patterns. Spatial normalization was performed to transform fMRI data from individual space to the University of North Carolina (UNC) brain standard space of 2‐year‐old (Shi et al., 2011) with a nonlinear registration method, using the Advanced Normalization Tools (ANTs, http://stnava.github.io/ANTs/).

### 2.3 Seed‐based FC map

**FIGURE 1:**
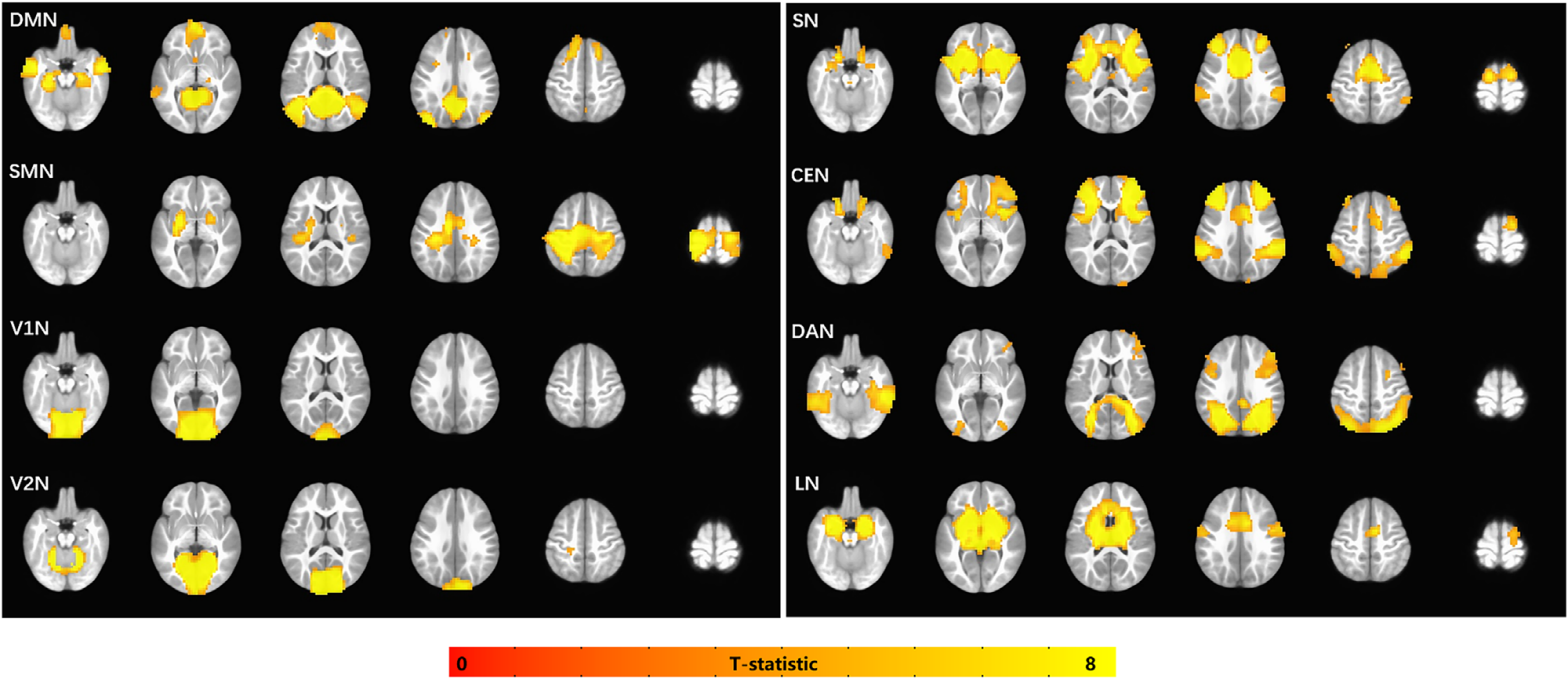
**Resting‐state functional network regions**. This picture shows the expanded seed regions of each functional network (cluster‐level multiple comparison correction at alpha < 0.05 with voxel‐level *p*‐value < .001 and cluster size > 24). DMN, default mode network; SMN, sensorimotor network; V1N, medial visual network; V2N, occipital pole visual network; SN, salience network; CEN, central executive network; DAN, dorsal attention network; LN, limbic network.

Network‐level FC analyses using seed‐based FC map were focused on seven formerly proposed intrinsic functional networks, which include visual network (VN), sensorimotor network (SMN), default mode network (DMN), salience network (SN), central executive network (CEN), dorsal attention network (DAN), and limbic network (LN) (Raichle, 2011; Smith et al., 2009; Yeo et al., 2011). Besides, VN was further divided into medial visual network (V1N) and occipital pole visual network (V2N). Networks were selected as a result of their association with the visual, sensorimotor, and neurocognitive aspects of IE patients (Guo, Chen, Huang et al., 2022a; Guo, Chen, Liu et al., 2022b). For V1N/V2N/SMN, a sphere with 6‐mm radius, centered at the global maximum of each network map (Smith et al., 2009), was defined as the seed region (Gao et al., 2013), which were located at the calcarine cortex, occipital pole, and precentral gyrus, respectively. For DAN/SN/CEN, a sphere with 6‐mm radius centered at MNI: −27 −52 57; 24 −56 55/MNI: −32 24 −10; 38 26 −10/MNI: −32 44 16; 44 36 20 (Gao & Lin, 2012; Seeley et al., 2007; Vincent et al., 2008), which were located at bilateral intraparietal sulcus, anterior insula, and dorsolateral prefrontal cortex. These sphere masks were spatially transformed to the UNC‐infant‐2‐year‐old space. For DMN and LN, the posterior cingulate cortex and amygdala from infant‐2‐year‐old atlas (Shi et al., 2011) were selected as seed regions. Individual‐level correlations of time series of each seed region with every other voxel across the brain were computed and Fisher *z* transformed in IE patients and healthy subjects. The mask of each functional network was generated in healthy controls with a series of expanded seed areas, where the voxels satisfy the significant positive connectivity (*Z* > 0 with voxel *p* < .001 and cluster size > 35) with the seed regions at the group level (Figure 1). Intranetwork (within each network map) or internetwork (out of each network map) FC was defined by these predefined group masks of intrinsic networks.

### 2.4 Statistical analysis

A linear mixed effects model (LME) was applied for group‐level FC contrasts, in which the age, gender, group, and the interaction items between age and group were taken as fixed effects while the random intercept and slope for group were regarded as random effects. Specifically, in the cross‐sectional network‐level FC analysis, we focused on the group (IE vs. controls) effect controlling the effects of age and gender, and additional group‐age interaction effect. In the longitudinal network‐level FC analysis, we attached importance to examine the group (preoperative group vs. postoperative group) effect, controlling the effects of age, gender, and timing of onset. The statistical significance threshold of *p* < .05 with cluster‐level correction (Alphasim correction, voxel‐level *p* < .001 with cluster > 24 voxels) was applied to reduce the false positives from multiple comparisons. Pearson correlation analysis was performed to assess the relationship between the altered FC and clinical variables in IE patients. Between‐group difference in demographics was examined using two‐tailed independent sample *t* test or chi‐square test. A *p*‐value of < 0.05 was considered for establishment of the statistical significance.

## 3 RESULTS

### 3.1 Demographics and clinical features

At the baseline fMRI scanning, 32 IE patients and 30 healthy participants had similar profiles in age (*p* = .306) and gender (*p* = .987) (Table 1). All 17 postoperative patients with IE restored the normal ocular alignments during the follow‐up period. Only nine IE patients finished the stereovision evaluation, among whom six patients were demonstrated the restoration of stereopsis in different degrees while the remaining patients failed to acquire any improvement in stereoscopic vision. Other children with IE were too young to cooperate well in this step, hence, the stereovision metrics of them were not available. See Table 2 for more detailed demographical and clinical information of patients with IE.

**TABLE 1:** Demographics and clinical characteristics of subjects at baseline.

	IEs (n = 32)	HCs (n = 30)	*p*‐value
Age (years)	3.00 ± 1.81	3.47 ± 1.76	.306^a^
Sex, male/female	17/15	16/14	.987^b^
Onset (months)	4.16 ± 1.78	N/A	N/A
Strabismus degree (PD)	53.41 ± 18.12	N/A	N/A

IEs, infantile esotropia patients; HCs, healthy controls; N/A, not available; PD, prism degree.

^a^ Two‐sample two‐tailed t‐test.

^b^ Chi‐square test.

**TABLE 2:** Demographics and clinical characteristics of patients with IE before and after surgery.

Subject	Sex	Age (years)		Onset (months)	Strabismus degree (PD)		Stereovision (+/‐)		Follow‐up period months)
		Presurgery	Postsurgery		Presurgery	Postsurgery	Presurgery	Postsurgery	
S1	M	6.36	7.11	5	30	<15	–	+	8.98
S2	M	2.50	3.02	6	45	<15	–	+	6.21
S3	M	1.68	2.98	3	90	<15	–	+	15.65
S4	F	1.76	2.77	3	70	<15	–	N/A	12.07
S5	M	2.20	3.38	1	40	<15	–	–	14.14
S6	M	6.15	6.90	5	20	<15	–	–	9.01
S7	M	5.19	5.76	6	40	<15	–	+	6.87
S8	M	0.73	1.87	3	50	<15	–	N/A	13.64
S9	F	6.16	7.35	3	70	<15	–	–	14.20
S10	M	1.17	2.06	5	70	<15	–	N/A	10.72
S11	F	1.73	2.67	2	75	<15	–	N/A	11.31
S12	M	0.41	1.27	3	60	<15	–	N/A	10.22
S13	F	2.22	3.48	4	70	<15	–	+	15.09
S14	F	2.69	4.01	1	75	<15	–	+	16.21
S15	F	0.53	1.40	3	40	<15	–	N/A	10.42
S16	M	1.25	2.16	6	40	<15	–	N/A	11.01
S17	M	0.92	2.17	4	55	<15	–	N/A	14.99
Average		2.57 ± 2.10	3.55 ± 2.00	3.71 ± 1.61	55.24 ± 19.02				11.81 ± 3.04

N/A, not available; PD, prism degree.

### 3.2 Cross‐sectional network‐level analyses

**FIGURE 2:**
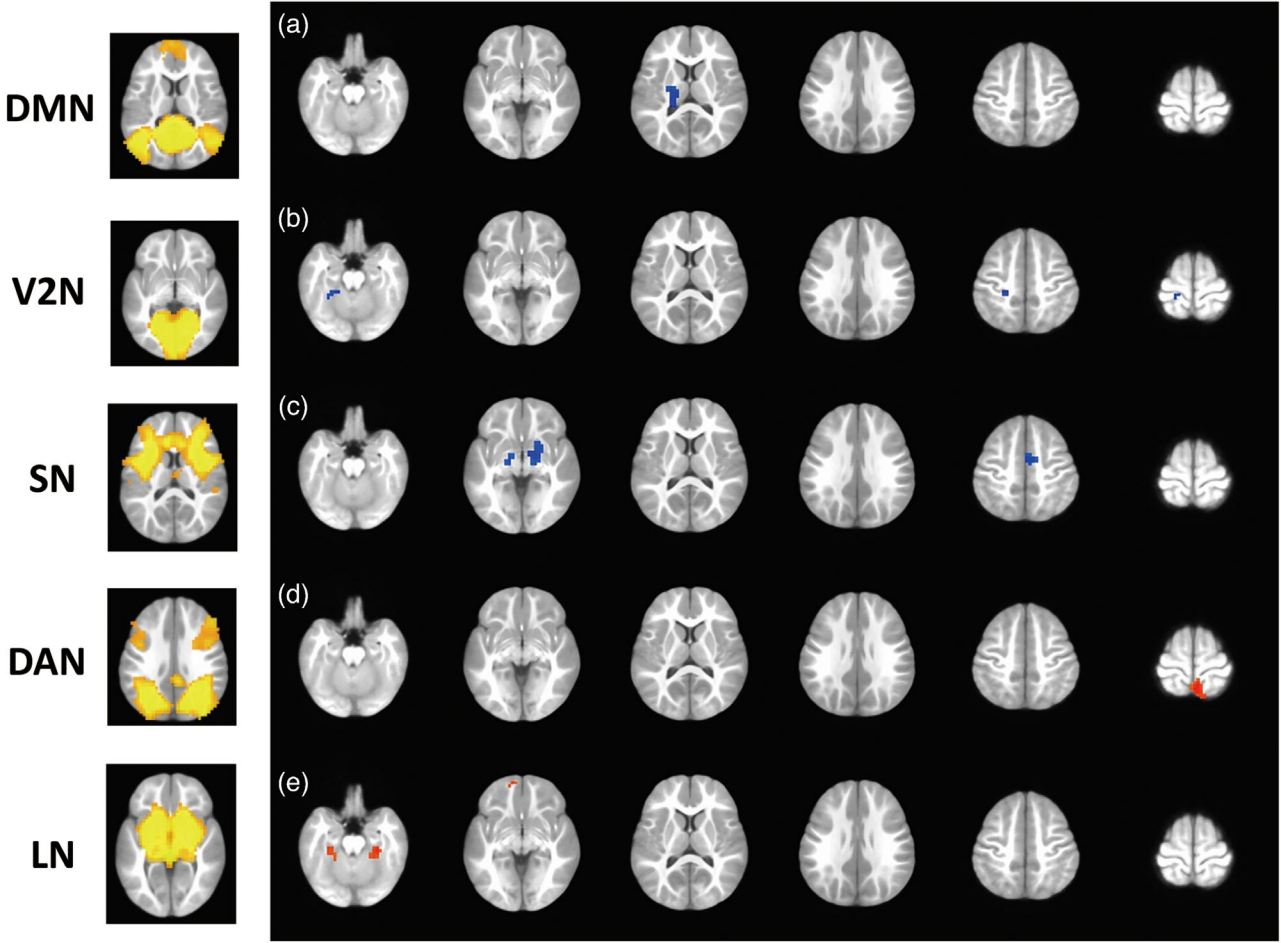
**Brain regions with significant between‐group difference in functional connectivity of different networks in cross‐sectional analysis**. (a) Decreased FC of default mode network with left thalamus; (b) decreased FC of occipital pole visual network with left postcentral gyrus and left fusiform gyrus; (c) decreased FC of salience network with right putamen nucleus, left hippocampus, right median cingulate and paracingulate gyri; (d) increased FC of dorsal attention network with right precuneus/right superior parietal gyrus; (e) increased FC of limbic network with bilateral fusiform gyri and left medial orbital part of superior frontal gyrus. Red: Infantile esotropia > healthy control; blue: infantile esotropia < healthy control. Cluster‐level multiple comparison correction at alpha < 0.05 with voxel‐level *p*‐value < .001 and cluster size > 24.

At baseline, the IE group exhibited significantly decreased network‐level FC between DMN and left thalamus (*p* = 1.37e‐06) of LN compared to healthy controls. Several lower network‐level FC were also demonstrated in IE patients, such as internetwork FC including V2N‐SMN (left postcentral gyrus; *p* = 4.08e‐05), V2N‐DMN (left fusiform gyrus; *p* = 7.21e‐06), and intranetwork FC of SN‐SN (right putamen nucleus, *p* = 2.91e‐06; left hippocampus, *p* = 5.11e‐06; right median cingulate and paracingulate gyri, *p* = 3.42e‐06). By contrast, higher network‐level FC was found in IE patients in DAN‐DMN (right precuneus/right superior parietal gyrus; *p* = 1.16e‐05) and LN‐DMN (right fusiform gyrus, *p* = 4.37e‐07; left fusiform gyrus, *p* = 1.34e‐05; left medial orbital part of superior frontal gyrus, *p* = 1.02e‐05) (Figure 2).

**FIGURE 3:**
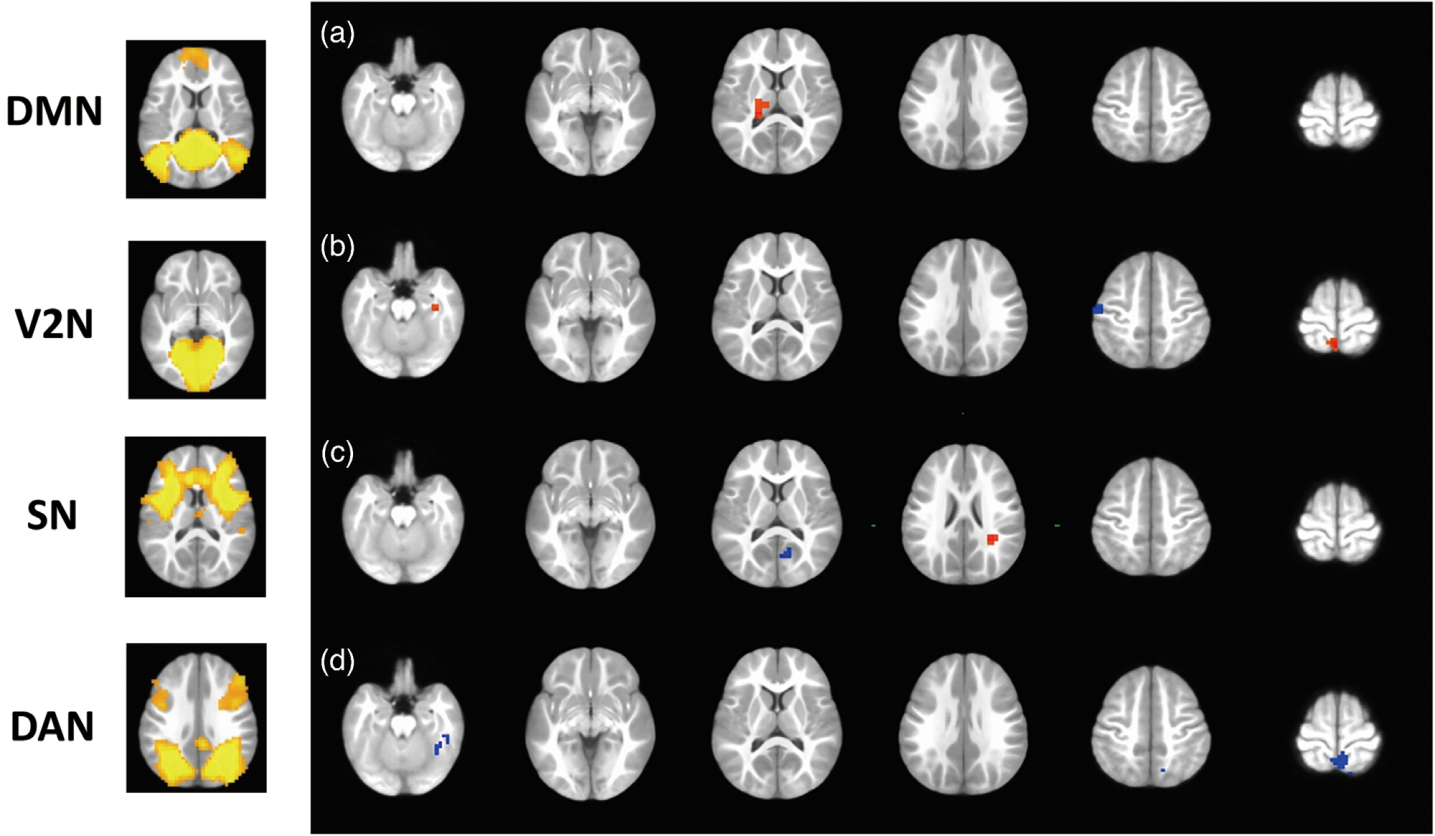
**Brain regions with significant group‐age interaction in functional connectivity of the default mode network (a), occipital pole visual network (b), salience network (c), and dorsal attention network (d)**. The functional connectivity of default mode network with left thalamus, occipital pole visual network with right inferior temporal gyrus, left precuneus, left postcentral gyrus, salience network with right inferior parietal lobule, right calcarine fissure and surrounding cortex, dorsal attention network with right precuneus/right superior parietal gyrus, and right inferior temporal gyrus/right fusiform gyrus exhibited significant interaction between group and age in the cross‐sectional analysis. Red: Functional connectivity increase with age in infantile esotropia; blue: Functional connectivity decrease with age in infantile esotropia. Cluster‐level multiple comparison correction at alpha < .05 with voxel‐level *p*‐value < .001 and cluster size > 24.

In addition, significant group‐age interactions were observed in such network‐level FC as DMN‐LN (left thalamus; *p* = 1.37e‐06), V2N‐DMN (right inferior temporal gyrus, *p* = 7.21e‐06; left precuneus, *p* = 7.21e‐06), V2N‐SMN (left postcentral gyrus, *p* = 7.21e‐06), SN‐DAN (right inferior parietal lobule; *p* = 3.42e‐06), SN‐DMN (right calcarine fissure and surrounding cortex; *p* = 3.42e‐06), DAN‐DMN (right precuneus/right superior parietal gyrus; *p* = 1.16e‐05), and intranetwork FC of DAN‐DAN (right inferior temporal gyrus/right fusiform gyrus; *p* = 1.16e‐05) (Figure 3; Supporting Information Table S1 for detailed clusters’ information).

### 3.3 Longitudinal network‐level analyses

**FIGURE 4:**
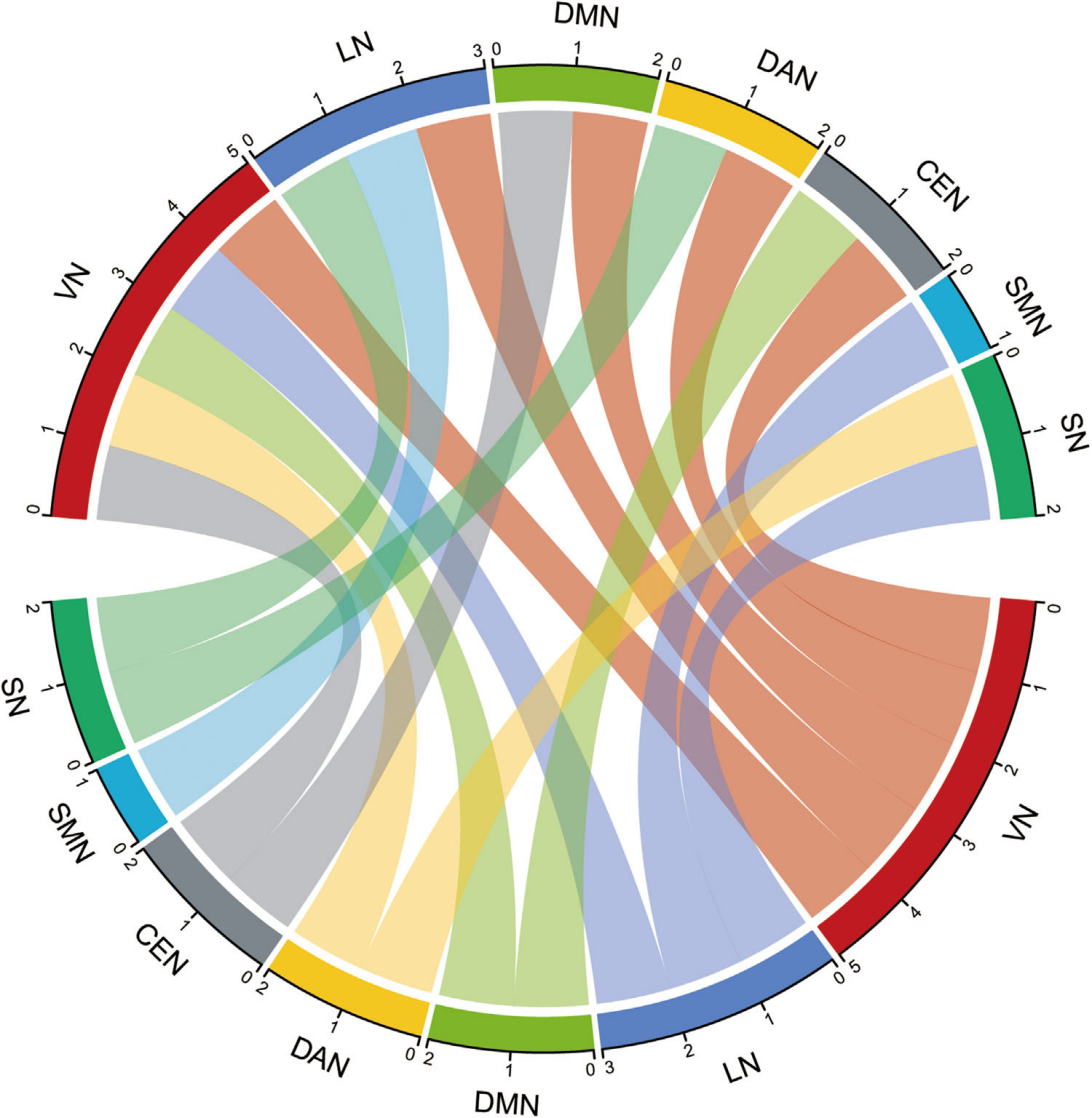
**Functional network connectivity with significant alterations in longitudinal analysis**. Network‐level functional connectivity of visual network changed widespreadly in postoperative patients with infantile esotropia. DMN, default mode network; SMN, sensorimotor network; VN, visual network; SN, salience network; CEN, central executive network; DAN, dorsal attention network; LN, limbic network.

**FIGURE 5:**
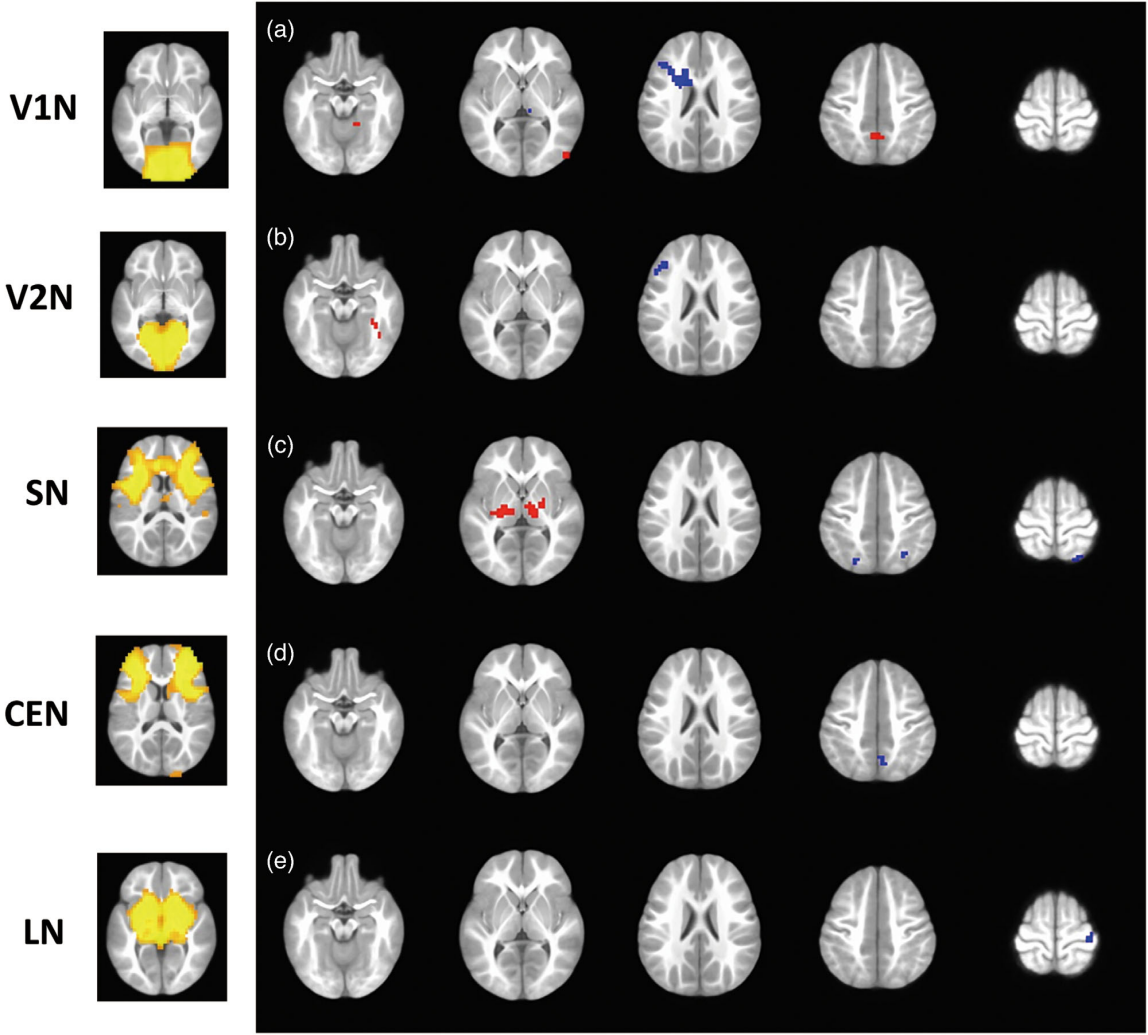
**Brain regions with significant alterations in functional connectivity of the medial visual network (a), occipital pole visual network (b), salience network (c), central executive network (d), and limbic network (e) in patients with infantile esotropia performed strabismus surgery**. The functional connectivity of medial visual network with left middle frontal gyrus/left precentral gyrus/left triangular part of inferior frontal gyrus, right middle temporal gyrus, right lingual gyrus and precuneus, occipital pole visual network with right fusiform gyrus and left middle frontal gyrus, salience network with bilateral thalami and bilateral superior parietal gyri, central executive network with right precuneus, and limbic network with right postcentral gyrus changed significantly in postoperative patients. Red: postoperative > preoperative; blue: postoperative < preoperative. Cluster‐level multiple comparison correction at alpha < .05 with voxel‐level *p* < .001 and cluster size > 24.

The LME was used to compare the functional network connectivity of IE patients between both fMRI scanning time points, controlling the effect of age, gender, and time of onset. Compared to preoperative IE patients, postoperative group showed a wide spread of altered network‐level FC of VN as well as the changed between‐network FC of LN, DAN, DMN, CEN, SN, and SMN (Figure 4). Specifically, alterations in internetwork FC in V1N‐LN (left middle frontal gyrus/left precentral gyrus/left triangular part of inferior frontal gyrus; *p* = 7.91e‐11), V1N‐DAN (right middle temporal gyrus; *p* = 7.95e‐05), V1N‐DMN (bilateral precuneus; *p* = 6.52e‐06), V2N‐DAN (right fusiform gyrus; *p* = 2.83e‐11), V2N‐CEN (left middle frontal gyrus; *p* = 5.37e‐06), SN‐LN (right thalamus, *p* = 2.33e‐05; left thalamus, *p* = 8.97e‐07), SN‐DAN (right superior parietal gyrus, *p* = 1.66e‐05; left superior parietal gyrus, *p* = 7.03e‐06), CEN‐DMN (right precuneus; *p* = 4.44e‐05), LN‐SMN (right postcentral gyrus; *p* = 4.70e‐05) as well as in intranetwork FC of V1N‐V2N (right lingual gyrus; 5.97e‐06) were evident in postoperative IE patients (Figure 5). Of note, patients with strabismus surgery showed decreased FC in V1N‐LN and V2N‐CEN. By contrast, increased FC was observed in postoperative IE patients in V1N‐DAN, V1N‐DMN, V2N‐DAN, and V1N‐V2N (Supporting Information Table S1 for detailed clusters’ information).

### 3.4 Correlation of longitudinal FC changes with baseline clinical metrics

**FIGURE 6:**
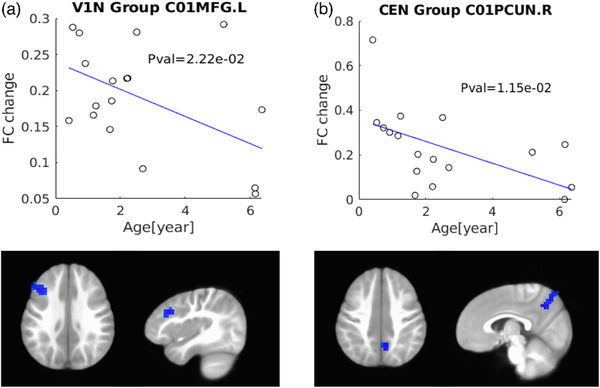
**Correlation of longitudinal functional connectivity changes with age at surgery**. The longitudinal FC changes in terms of V1N‐LN (left middle frontal gyrus) (a) and CEN‐DMN (right precuneus) (b) negatively associated with the age at surgery in patients with infantile esotropia. CEN, central executive network; DMN, default mode network; V1N, medial visual network; LN, limbic network; FC, functional connectivity; PCUN, precuneus; MFG, middle frontal gyrus; R, right; L, left.

Significant negative correlations of the age at surgery with longitudinal FC changes in terms of CEN‐DMN (right precuneus; *p* = 1.15e‐02) and V1N‐LN (left middle frontal gyrus; *p* = 2.22e‐02) were observed in patients with IE (Figure 6). There was no evident correlation between longitudinal FC alterations and baseline strabismus degree in IE.

## 4 DISCUSSION

Here, we examined alterations of large‐scale functional network connectivity in IE patients using cross‐sectional samples as well as longitudinal data, and discovered robust evidence that patients with IE present abnormal FC within or between large‐scale functional networks (i.e., VN, SMN, DMN, SN, CEN, DAN, and LN) compared to healthy subjects. Significant group‐age interactions were also observed for network‐level FC, suggesting aberrant FC development trajectories in IE patients, which are consistent with our previous findings (Guo, Chen, Liu et al., 2022a). Additionally, patients with IE who underwent corrective surgery exhibited significant large‐scale functional network connectivity changes following an approximately 1‐year follow‐up period. Furthermore, longitudinal FC changes in terms of CEN‐DMN and V1N‐LN negatively correlated to the age at surgery in IE. As no previous researches have assessed whether corrective surgery does exert an effect on the brain function in IE, our longitudinal data, for the first time, lend support to the presence of a changed large‐scale network FC in postoperative patients with IE.

### 4.1 Cross‐sectional network‐level FC alterations in IE

Majority of the studies on resting‐state FC, in the context of strabismus, laid importance on V1‐based FC analyses, with similar findings of deficient interactions between visual cortex and brain regions pertaining to stereopsis or eye movement (He et al., 2021; Liu et al., 2022; Yan et al., 2019; Zhu et al., 2018). Attaching importance to intra‐ and internetwork connectivity, instead of regional or global FC, suggests an advance on preceding studies, particularly in view of prominent evidence of disrupted between‐network FC in patients with IE (Guo, Chen, Liu et al., 2022a).

The VN‐SMN internetwork FC is disrupted in IE patients, of which the defects occur in the early postnatal period and persist across the infancy and early childhood, showing a characteristic developmental trajectory with age. The interaction between VN and SMN is implicated in visuomotor coordination (Burgos et al., 2018; Hou et al., 2022), thus, impairment in VN‐SMN internetwork FC could account for the observed hand‐eye coordination disorder in IE patients. As a higher‐order functional network, SN is considered to be implicated in integrating external and internal information, attaching salience weighting to different events, regulating the response of other networks to external stimulus or internal physiological signal, coordinating the correlation between functional networks (e.g., DAN‐DMN) (Gao et al., 2013; Gao et al., 2017; Seeley et al., 2007). The FC between SN and subcortical structure was reported to be capable of predicting the visuospatial working memory performance and cognitive function (Alcauter et al., 2014). Furthermore, the FC between SN and limbic regions plays a critical role in the regulation of emotion, reward, and motivation processing (Seeley et al., 2007). Taken together, impaired network‐level FC between SN and those networks involved in visual attention (e.g., DAN), cognition and emotion (e.g., DMN), coupled with abnormal FC within SN and disrupted interaction between DAN and DMN, indicate the disorder of SN serving as “regulators,” which may contribute to the disturbance of visual function, neurocognitive development, and emotional management in IE patients. The FC between DMN and thalamus (i.e., LN) emerges during the infancy and involves self‐concept construction (Philippi et al., 2012). Anomalous DMN‐thalamus FC further provides neurobiological evidence for the potential cognitive processing impairment in patients with IE.

Our results indicate that, in IE, different network‐level FC are not equally interfered in developmental trajectories. More interestingly, internetwork FC are more prone to be interfered than intranetwork ones. One possible explanation is that primitive functions based on single brain network mature very early after birth to guarantee survival, whereas complicated and high‐order functions relying on internetwork interactions require prolonged environment‐related tuning and, thus, are vulnerable to abnormal visual experience (Gao et al., 2015).

### 4.2 Longitudinal network‐level FC changes in IE

The VN is responsible for broadly categorized visual information processing such as the visual content information and the visuospatial information (Yamamoto et al., 2012). Here, the obviously altered functional network connectivity within VN might play a pivotal role in the improvement of binocularity in postoperative IE patients. Recently, a longitudinal resting‐state MRI study also demonstrated significant functional changes in core regions of VN in strabismus patients with binocularity recovery following corrective surgery (Wu et al., 2022). In this study, the VN‐DMN internetwork FC was decreased in the preoperative IE patients but significantly increased in the postoperative counterparts. VN‐DMN internetwork FC was reported to be involved in social visual engagement behaviors, the impairment of which contributes to ignoring some specific kinds of input information from environment to brain and results in social communication difficulties (Lombardo et al., 2019). Thus, the improvement of VN‐DMN may be important for IE patients in the development of social communication. In addition, the alterations of network‐level FC between VN and several networks (i.e., DAN, LN, and CEN) were also detected in postoperative IE patients. The DAN is related to visuospatial attention, eye movement, and visuomotor coordination (Corbetta & Shulman, 2002). Alterations of DAN‐VN internetwork FC may contribute to ameliorative top‐down control of visual attention in IE patients with corrective surgery, and thus, are beneficial for the hand‐eye coordination. CEN is another higher‐order control network that regulates the response of other networks (e.g., VN, SMN, and LN) to external stimulus and cooperates with those networks in processing external sensory input. Additionally, CEN is involved in visual attention regulation and decision making (Miller & Cohen, 2001). Therefore, CEN‐VN internetwork FC varying longitudinally is conducive to the external visual information processing and may facilitate the cognition development or observed stereovision improvement of postoperative IE patients. Although “competing” mechanisms between CEN and DMN are obvious in some context, they are also closely coupled and are coincidentally anomalous in depression (Chang & Glover, 2009; Fox et al., 2005; Liston et al., 2014). Thus, altered CEN‐DMN internetwork FC may lead to the improvement of the emotion regulation of IE patients. The LN is dedicated to a series of processes, including memory consolidation, integration of visceral sensory information with behaviors, emotion modulation, and visuospatial orientation (Catani et al., 2013; Vann et al., 2009). In this study, the LN displayed altered FC with such crucial networks such as VN, SMN, and SN among postsurgical children with IE, which may exert a favorable effect on their developing brain and contribute to their visual, sensorimotor, and cognitive function recovery.

Interestingly, in this study, we demonstrated apparent negative correlations of the age at surgery with longitudinal FC changes in terms of CEN‐DMN and V1N‐LN, suggesting that the earlier the strabismus surgery is performed, the more benefits the IE patients will obtain in visual processing or emotion regulation, which emphasizes the importance of the timely intervention for IE patients. More importantly, the findings provide robust evidence for our previous hypothesis from cross‐sectional studies that corrective surgery for IE patients should be implemented in the early postnatal period to help the affected children to approach the normal developmental trajectories in FC (Guo, Chen, Liu et al., 2022a).

### 4.3 Limitations

Several limitations should be considered when illustrating the findings. First of all, due to the young age of the IE patients enrolled in this study, postoperative stereovision evaluation was only performed in those capable of subjectively cooperating in the Titmus stereogram examination. As a result, available quantified stereopsis data were not sufficient in the correlation analysis with network‐level FC alterations. Besides, our research with a relatively small sample size and future work with a larger number of subjects in both cross‐sectional and longitudinal sample, coupled with multiple follow‐up time points, are necessary to note at which time the process of stereovision improvement begins following the corrective operation. Furthermore, such further researches should also acquire estimates of cognitive function based on assessment scales to better delineate the interaction between network‐level connectivity alteration and cognition function restoration in IE. Also of note, different depths of sleep may exert effect on the properties of network or functional network connectivity. However, it is not easy to carry out overt monitoring of sleep status employing electrophysiological methods in this population (Liu et al., 2021). Future studies would be performed to try to compare the network connectivity under different sleep stages. By generating masks based on the healthy controls alone, we aimed to capture the functional network patterns specific to the healthy population, producing typical masks of functional networks for group‐level comparison, however, this may introduce group‐specific confounding effects in this study.

## 5 CONCLUSIONS

In conclusion, patients with IE exhibit significantly impaired FC between or within large‐scale functional networks, which are implicated in cognitive, emotional, visual, and sensorimotor processing. Alterations of intra‐ or internetwork FC of those networks in postoperative IE patients render new insights into the neurobiological process underlying the observed functional improvements in terms of stereoscopic depth vision, hand‐eye coordination, and emotional regulation. Moreover, the internetwork FC is more prone to be disturbed under the influence of abnormal visual input in IE. More importantly, the corrective surgery for IE patients should be performed as early as possible to help them to obtain more benefits in visual‐ or emotional‐related brain functions. On the basis of our results, future explorations with preclinical purposes, such as predicting surgical benefits or discovering biomarkers for predicting stereopsis restoration for IE, would be conducted to attain more meaningful and practicable achievements for clinical practice.

## AUTHOR CONTRIBUTIONS

YP, NL, and DH designed the study. JG was in charge of acquiring imaging data and drafting the manuscript. WL, LH, YL, and HK contributed to recruiting participates and collecting clinical data. YC was responsible for data analysis. All authors approved the final manuscript.

## CONFLICT OF INTEREST STATEMENT

The authors declare no conflict of interest.

### PEER REVIEW

The peer review history for this article is available at https://publons.com/publon/10.1002/brb3.3154.

## Supporting information

Supplementary Information

## Data Availability

Data available on request from the authors
