# Supplementary material for: Alterations of large‐scale functional network connectivity in patients with infantile esotropia before and after surgery
Source: Brain Behav. 2023 Jul 11;13(8):e3154. doi: 10.1002/brb3.3154 (PMC10454265; doi:10.1002/brb3.3154)
Supplement: Supplementary file 1 — Supplementary Information [file BRB3-13-e3154-s001.pdf]

**Supplementary Table 1**

**Clusters' information of group effects**

| | Peak Location in standard space | Peak T statistic | No. of Voxels | Regions (2year-old-AAL) |
|---|---|---|---|---|
| **V2N** | | | | |
| 1 | [20 32.8 50.5] | -4.82 | 32 | PoCG.L |
| 2 | [27 22.2 -30] | -5.24 | 29 | FFG.L |
| **SN** | | | | |
| 1 | [-18.5 -9.2 -9] | -4.97 | 74 | PUT.R |
| 2 | [-4.5 4.8 40] | -5.45 | 45 | HIP.L |
| 3 | [16.5 11.8 -9] | -5.13 | 45 | DCG.R |
| **DAN** | | | | |
| 1 | [-4.5 53.8 57.5] | 4.69 | 64 | PCUN.R; SPG.R |
| **DMN** | | | | |
| 1 | [20 25.8 12] | -5.16 | 34 | THA.L |
| **LN** | | | | |
| 1 | [-18.5 25.8 -16] | 5.12 | 48 | FFG.R |
| 2 | [27 22.2 -19.5] | 4.90 | 33 | FFG.L |
| 3 | [9.5 54.8 -9] | 4.65 | 25 | ORBsupmed.R |

**Clusters' information of interaction effects of Age*Group**

| | Peak Location in standard space | Peak T statistic | No. of Voxels | Regions (2year-old-AAL) |
|---|---|---|---|---|
| **DMN** | | | | |
| 1 | [20 25.8 12] | 4.84 | 24 | THA.L |
| **V2N** | | | | |
| 1 | [-39.5 -2.2 -37] | 4.55 | 41 | ITG.R |
| 2 | [2.5 53.8 57.5] | 4.90 | 32 | PCUN.L |
| 3 | [51.5 15.2 47] | -4.40 | 24 | PoCG.L |
| **SN** | | | | |
| 1 | [-29 39.8 22.5] | 4.45 | 37 | IPL.R |
| 2 | [-8 57.2 8.5] | -4.38 | 25 | CAL.R |
| **DAN** | | | | |
| 1 | [-1 53.8 54] | -4.60 | 80 | PCUN.R; SPG.R |
| 2 | [-32.5 39.8 -16] | -4.24 | 35 | ITG.R; FFG.R |

**Clusters' information of longitudinal effects**

| | Peak Location in standard space | Peak T statistic | No. of Voxels | Regions (2year-old-AAL) |
|---|---|---|---|---|
| **V2N** | | | | |
| 1 | [-32.5 46.8 -23] | 9.55 | 63 | FFG.R |
| 2 | [34 -26.8 29.5] | -5.00 | 45 | MFG.L |
| **SN** | | | | |
| 1 | [22 4.8 5] | 4.96 | 64 | THA.R |

| | | | | |
|---|---|---|---|---|
| 2 | [-29 64.2 50.5] | -5.38 | 63 | SPG.R |
| 3 | [23 18.8 5] | 5.07 | 43 | THA.L |
| 4 | [27 71.2 50.5] | -5.13 | 35 | SPG.L |
| **LN** | | | | |
| 1 | [-32.5 29.2 50.5] | -7.8 | 38 | PoCG.R |
| **CEN** | | | | |
| 1 | [-4.5 53.8 36.5] | -4.97 | 40 | PCUN.R |
| **V1N** | | | | |
| 1 | [34 -19.8 29.5] | -6.19 | 178 | MFG.L; PreCG.L; IFGtri.L |
| 2 | [-43 67.8 5] | 4.33 | 38 | MTG.R |
| 3 | [2.5 50.2 33] | 4.47 | 34 | PCUN |
| 4 | [-18.5 36.2 -12.5] | 4.70 | 26 | LING.R |
